# Hickam’s Suicide: A Case of Carbon Monoxide Toxicity, Compartment Syndrome, Rhabdomyolysis, and Renal Failure From Attempted Dual Suicide

**DOI:** 10.7759/cureus.46759

**Published:** 2023-10-09

**Authors:** Rachel E Bridwell, Richard R Miles, Sean Griffiths, Robert R Burgin, Brit Long

**Affiliations:** 1 Emergency Medicine, Madigan Army Medical Center, Joint Base Lewis McChord, USA; 2 Prehospital Services, US Military, Williamsburg, USA; 3 Emergency Medicine, Brooke Army Medical Center, Fort Sam Houston, USA; 4 Emergency Medicine, Uniformed Services University of the Health Sciences, Bethesda, USA

**Keywords:** dual suicide, rhabdomyolysis, compartment syndrome, aborted attempt, cyanide, carbon monoxide

## Abstract

Suicide pacts among elderly couples afflicted by a terminal disease process present a significant challenge to emergency clinicians. If one member of the pair aborts their attempt, the surviving member of a dual suicide attempt can present a complex case with numerous clinical issues reflected by Hickam’s dictum rather than by Occam’s razor. Thus, emergency clinicians must keenly search for a multitude of concomitant but compounding conditions, potentially projected onto pre-existing comorbidities in an elderly population. The authors present a case of a suicide pact in which one member of the couple completed the attempt while the surviving member experienced carbon monoxide toxicity, compartment syndrome, rhabdomyolysis, and renal failure following her aborted suicide attempt.

## Introduction

Suicide attempts are on the rise in all populations, particularly in the elderly, and the risk of attempted suicide is higher in patients with a diagnosis of cancer with poor prognosis [1]. Suicide pacts, when couples attempt suicide together, is a known phenomenon among the elderly, even when only one of the individuals has a poor prognosis [2-4]. However, if the healthier partner aborts or survives the initial suicide attempt, this insult may still result in significant morbidity and mortality [4]. These aborted suicide attempts can result in life-threatening conditions, marked not by Occam’s razor, in which a single diagnosis accounts for all symptoms, but by Hickam’s dictum, which states “a man can have as many diseases as he damn well pleases” [5]. The authors present a case of an elderly suicide pact suicide wherein one partner of the couple completed the attempt while the partner who halted their attempt suffered multifactorial critical illness.

## Case presentation

A previously healthy 72-year-old female with diet-controlled hyperlipidemia was transported by ambulance to a rural emergency department (ED) for an aborted suicide attempt in the Pacific Northwest during the winter. First responders found the patient on the floor of her sealed garage, which had both a barbecue burning unknown substances as well as a car engine running. Her husband, who had known terminal cancer, had expired in the garage at the scene. Paramedics found no other substances or paraphernalia at the scene, but two suicide notes were found, signed by the patient and her husband, respectively. The patient was initially hypoxemic on scene with an oxygen saturation of 73% but improved with a 15-liter (L) nonrebreather (NRB) mask to an oxygen saturation of 95%.

**Figure 1:**
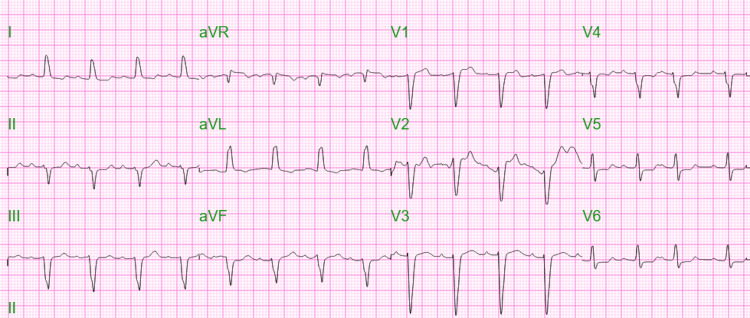
Electrocardiogram demonstrating sinus rhythm with a left bundle branch block

**Figure 2:**
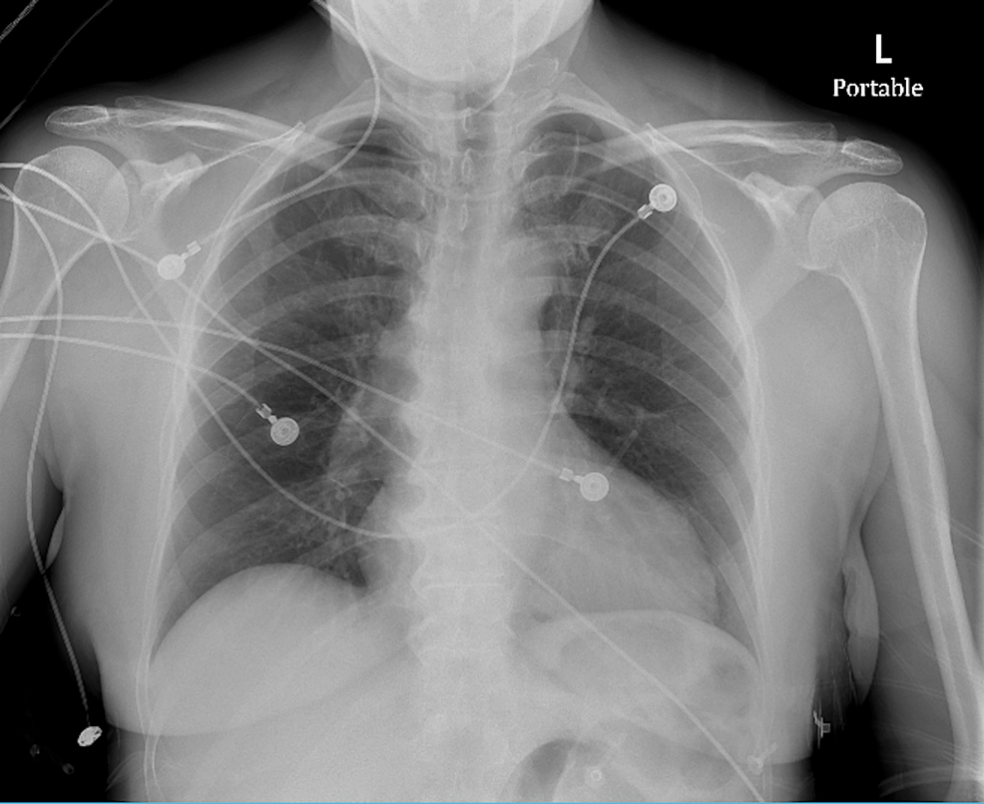
Anteroposterior chest radiograph demonstrating no acute intrathoracic pathology

On arrival to the ED, initial vital signs included oxygen saturation of 98% on 15 L NRB, heart rate of 96 beats per minute, respiratory rate of 31 breaths per minute, blood pressure of 81/59 mmHg, and rectal temperature of 93.4F. Physical examination was notable for a hypothermic female, alert but oriented only to place and time though she could not provide any insight or information into the suicide pact. The patient was also noted to have significant weakness in the right foot, was cool to the touch, with poor but severely painful dorsiflexion and plantar flexion. There were no other signs of external trauma. Laboratory evaluation demonstrated a white blood cell count of 21,400 cells/uL, carboxyhemoglobin level of 5.8%, lactate of 9.5 mmol/L, potassium of 7.6 mEq/L, creatinine of 2.46 mg/dL, aspartate transaminase (AST) of 4,947 U/L, alanine transaminase (ALT) of 1,717 U/L, troponin I of 17,983 ng/L, N-terminal pro-B-type natriuretic peptide (NT-proBNP) of 12,769 pg/mL, phosphorus of 7.3 mg/dL, 3+ blood on urinalysis without erythrocytes on microscopy, and creatine kinase (CK) of 292,190 U/L. Urine drug screen, ethanol, salicylate, and acetaminophen levels were unremarkable. The initial electrocardiogram (ECG) revealed sinus rhythm with a left bundle branch block and a first-degree atrioventricular block, a heart rate of 92 beats per minute without a previous ECG available for comparison (Figure 1). A single anteroposterior chest radiograph was unremarkable without evidence of infiltrate, pulmonary edema, or pleural effusion (Figure 2). The patient was rewarmed with a Bair hugger to 97.1F, received a 30 mL/kg bolus of lactated ringers, and was started on a 6L nasal cannula with 15L NRB for the treatment of suspected carbon monoxide (CO) toxicity. Based on the patient’s elevated lactate, hydroxocobalamin was empirically administered to treat cyanide (CN) toxicity. Despite the fluid resuscitation, the patient’s mean arterial pressure remained below 65 mmHg, and a norepinephrine infusion was initiated through a right triple lumen central venous catheter with a maximum dose of 15 mcg/min in addition to continued fluid infusion for rhabdomyolysis. Based on the patient's pain with passive range of motion and pain out of proportion to the exam, the right lower extremity was elevated due to suspected compartment syndrome.

Consultation with poison control and hyperbaric medicine recommended continued supplemental oxygen, although the patient was not a candidate for hyperbaric oxygen due to hemodynamic instability. The patient was transferred to a tertiary referral center for intensive care treatment. The patient was unresponsive to initial diuretics and was subsequently dialyzed for the first 14 days of her hospital stay. Her echocardiogram on arrival demonstrated a reduced ejection fraction of 37%. She underwent a four-compartment right leg fasciotomy on hospital day 1, complicated by acute venous and arterial occlusion of the right lower extremity on postoperative day 7 as well as pneumonia, for which she received a seven-day course of cefepime. After her 52-day hospital stay, her repeat echocardiogram showed an improved ejection fraction of 41-50%. She was cleared by psychiatry for discharge to a skilled nursing facility with residual peroneal weakness in her right foot.

## Discussion

Suicide pacts bring the potential for one individual to abort during the attempt, begetting serious medical implications and pathophysiologic derangements. While elderly dual suicides often involve CO toxicity, the presenting signs and symptoms of CO toxicity are nonspecific, including headache, nausea, and vomiting [6]. CO toxicity accounts for approximately 800 deaths in the U.S. annually, though this is likely an underestimation due to the non-specific signs and symptoms [7]. Management includes supplemental oxygen to displace CO from hemoglobin, which binds with 200 times greater affinity compared to oxygen [7]. While there is insufficient evidence to currently support the use of hyperbaric oxygen in CO toxicity, toxicologic and hyperbaric consultation should be considered if there is a loss of consciousness, new neurologic deficits or mental status change, end-organ ischemia or damage, or if the patient is pregnant; however, hyperbaric oxygen treatment can be challenging with critically ill patients due to their hemodynamic instability and high resource utilization within a confirmed chamber [6,8]. Additionally, gasoline exposure may carry deleterious effects due to the volatile agents acting as both central nervous system depressants as well as cardiac myocardial irritants [9].

Due to the combustion of various household materials, CN toxicity is common in patients with CO toxicity, likely secondary to other materials combusted (e.g., items burned on the barbecue in the described case). CN halts adenosine triphosphate (ATP) production and aerobic metabolism by inhibiting cytochrome oxidase a3 of complex IV [10]. The symptoms of CN toxicity may be non-specific, including headache, confusion, and decreased level of consciousness, though tachypnea, hypotension, tachycardia, dysrhythmia, and seizure can occur [11]. Serum lactate greater than 8 mmol/L has a 94% sensitivity for the diagnosis of CN toxicity [12]. The mainstay of treatment includes hemodynamic support and hydroxocobalamin 5 g IV, which demonstrates an improved return to baseline MAP in animal models as compared to sodium nitrite and sodium thiosulfate while avoiding the risk of methemoglobinemia as sodium nitrite and sodium thiosulfate increase the risk of this hemoglobinopathy [13]. Similar to CO toxicity, consultation with a toxicologist is helpful in patients with CN toxicity.

CO and CN toxicity do not fully account for the underlying pathology within patients who abort their suicide attempts. In addition to the actual method of suicide attempt, the consequences of these intended symptoms often require additional critical care. Within the above case, the patient’s physiological issues were best explained not by Occam’s razor but by Hickam’s dictum. Due to both the combustion of hydrocarbons and plastics as well as the unknown time of immobilization, the patient was initially suffering from CO and CN toxicity in addition to rhabdomyolysis complicated by electrolyte derangements, acute renal failure, and compartment syndrome; her significantly reduced glomerular filtration rate secondary to rhabdomyolysis decreases peripheral perfusion to extremity compartments as well as increases the level of circulating fibrinogen, factor VIII, and factor IX, increasing the patient's risk of compartment syndrome and vessels thrombosis, respectively. She later developed acute heart failure with reduced ejection fraction as well as acute venous and arterial occlusions. Based on cardiology consultation at the receiving facility, this was not thought to be secondary to Takotsubo cardiomyopathy. With the complexity of these various but simultaneous pathologies, emergency clinicians must maintain a broad differential to encompass the multifactorial conditions that may occur within this subset of patients.

## Conclusions

Emergency clinicians face significant clinical challenges from elderly dual suicides, leading to a critical patient with multiorgan failure and profound long-term sequelae. In these pairs, at least one of the partners usually has preexisting conditions with a poor prognosis and quality of life, especially in elderly patients with cancer. This case underscores the multitude of life-threatening issues for those patients who do not complete their attempt, creating a myriad of clinical challenges. Emergency clinicians should be aware of these patients, mobilizing all specialty resources early, especially in resource-limited environments.
